# Phosphatidylethanol in Maternal or Neonatal Blood to Detect Alcohol Exposure during Pregnancy: A Systematic Review

**DOI:** 10.3390/life12101528

**Published:** 2022-09-30

**Authors:** Lisa Franceschetto, Matteo Perilli, Alessandro Cinquetti, Chiara Giraudo, Mario Gardi, Giovanni Cecchetto, Guido Viel

**Affiliations:** 1 Legal Medicine and Toxicology, Department of Cardiac, Thoracic, Vascular Sciences and Public Health, University of Padova, Via G. Falloppio 50, 35121 Padova, Italy; 2 UOSD Imaging Avanzato Clinico e Translazionale, Department of Medicine, University of Padova, 35127 Padova, Italy; 3 Unit of Urology, Sant’Antonio Hospital, University Hospital of Padua, 35100 Padua, Italy

**Keywords:** prenatal alcohol exposure, pregnancy, fetal alcohol spectrum disorders, phosphatidylethanol (PEth)

## Abstract

Background: Alcohol consumption during pregnancy, even at low doses, may damage the fetus. Pregnant women tend to underreport their alcohol consumption generating the need for sensitive and specific biomarkers, among which PEth has emerged due to its high specificity and possibility to be measured in both maternal and neonatal blood. The aim of this study is to systematically review the latest 20 years of literature for depicting the state of the art, the limitations, and the prospects of PEth for estimating alcohol consumption during pregnancy. Materials and methods: A systematic search, adhering to PRISMA guidelines, of the latest 20 years of literature through “MeSH” and “free-text” protocols in the databases PubMed, SCOPUS, and Web of Science, with time limits 1 January 2002–1 March 2022, was performed. The inclusion criteria were as follows: PEth used for detecting alcohol consumption during pregnancy, quantified in blood through liquid chromatography coupled to mass spectrometry, and full texts in the English language. Opinion papers, editorials, and narrative reviews were excluded. Results: Sixteen (16) papers were included in the present review (0.81% of total retrieved records). All the included records were original articles, of which there were seven prospective cohort/longitudinal studies, six cross-sectional studies, two observational-descriptive studies, and one retrospective study. All studies assayed PEth in at least one biological matrix; seven (7) studies quantified PEth in maternal blood, seven studies in newborn blood, and only two studies in both maternal and neonatal blood. In several included papers, PEth proved more sensitive than self-reports for identifying pregnant women with an active alcohol intake with the diagnostic efficiency improving with the increase of the maternal alcohol intake. Conclusions: Further studies, performed on wider and well-stratified populations, are needed to drive any definitive conclusion. PEth is a promising marker for monitoring alcohol use in pregnancy; however, at the present time, its use is still limited mainly by the absence of a globally agreed interpretative cut-off, the paucity of data regarding its specificity/sensitivity, and the lack of standardization on the diagnostic efficiency of the different isoforms.

## 1. Introduction

Prenatal alcohol exposure (PAE) can cause birth defects and lifelong neurocognitive deficits in affected children collectively termed “Fetal Alcohol Spectrum Disorders” (FASD) [1,2]. One of the ongoing challenges for an early and accurate diagnosis of FASD is the difficulty of assessing whether a mother drank alcohol during her pregnancy [3].

Alcohol can cross the placenta, with the fetal blood alcohol level tending to become similar to the maternal one, with a prolonged exposure due to slower elimination and accumulation in the amniotic fluid, which is swallowed by the fetus [4]. Although little is known about the effects of low levels of maternal alcohol intake on the neuropsychological development of the fetus and the child, as alcohol is neuro-teratogenic interacting with molecular regulators of brain development, any alcohol intake during pregnancy should be considered unsafe [5,6,7]. Hence, there is an importance of identifying women who are exposed to alcohol during pregnancy in order to educate them on the potential dangers for the fetus and provide counseling to reduce the risk of developing alcohol use disorders. The most used method for estimating alcohol use during pregnancy remains “self-report” through unstructured or structured questionnaires (e.g., TWEAK or AUDIT-C), but women tend to underreport their alcohol consumption out of shame or social stigma [8]. Thus, there is an importance of seeking biomarkers that are both sensitive and specific for identifying women who use alcohol during pregnancy, especially during the first trimester, which is considered the period of higher susceptibility of the fetus [9].

Alcohol is highly metabolized in the liver via the oxidative pathway (95%), while the remaining 5% is metabolized via the nonoxidative pathway in the pancreas, liver, brain, heart, and other organs [10]. All these metabolites could serve as potential direct biomarkers of alcohol consumption. However, an “ideal biomarker” should exhibit high sensitivity and high specificity, being related to the amount of alcohol consumed, allowing to reconstruct the pattern of consumption and the time-window of alcohol exposure [11].

Beside direct biomarkers, also indirect biomarkers, which become measurable in the body due to the toxic effect of alcohol on organs and systems, have been reported in the literature. These include, for example, serum liver enzymes such as γ-glutamyl transferase and mean corpuscular volume (MCV) of red blood cells [12].

Among direct alcohol biomarkers, which are generally more specific than indirect biomarkers, the most used ones are: Fatty Acid Ethyl Esters (FAEEs), Ethyl Glucuronide (EtG), Ethyl Sulfate (EtS), and Phosphatidylethanol (PEth) [13]. The latter is an abnormal cellular membrane phospholipid of red blood cells formed via a non-oxidative pathway of ethanol through the action of phospholipase D [14].

PEth is measured in blood and can identify even low levels of alcohol consumption over an extended period of time [15], with an average detection window of about 2 weeks after discontinuing alcohol consumption in alcoholic patients [16]. PEth can be used in several clinical and forensic settings, for confirming abstinence, detecting social drinking, or diagnosing alcohol related disorders, such as chronic excessive drinking, alcohol abuse, or dependence. It has recently been proposed also for identifying and monitoring alcohol intake in pregnant women, which is the subject of this review. Alcohol exposure during pregnancy can also be detected in the newborn immediately after birth, for example, quantifying the biomarker FAEE in the meconium (i.e., FAEE in the meconium is currently the most employed diagnostic test for PAE) [17].

More recently, PEth has received great attention in this field as it can be measured both in maternal blood during pregnancy and in neonatal blood after birth (also using capillary blood spots). Indeed, PEth can be quantified through liquid chromatography coupled to mass spectrometry (LC-MS) both in liquid and dry media, such as dried blood spots (DBS), which display ease of collection, storage, and transportation, maintaining the diagnostic efficiency of liquid blood [18].

In light of the above, the aim of this paper is to perform a systematic review of the latest 20 years of literature for depicting the state of the art, the limitations, and the prospects of PEth for estimating alcohol consumption during pregnancy.

## 2. Materials and Methods

This systematic review was carried out following the criteria included in the 2020 Preferred Reporting Items for Systematic Reviews and Meta-Analyses (PRISMA) guide [19].

In March 2022, one author (LF) performed a systematic search of the literature through “MeSH” and “free-text” protocols in the databases PubMed, SCOPUS, and Web of Science, with time limits 1 January 2002–1 March 2022; the following search terms were utilized for PubMed and Web of Science: (“Phosphatidylethanol” [Supplementary Concept] OR PEth OR Phosphatidylethanol) AND (forensic OR legal OR biomarker OR marker OR alcohol abuse OR abstinence OR monitoring”), while a slightly modified search string was used for Scopus: “ALL ((Phosphatidylethanol OR PEth) AND (forensic OR legal OR biomarker OR marker OR alcohol AND abuse OR abstinence OR monitoring))”. Paper selection was conducted independently by three authors (LF, AC and MP), based on titles and abstracts. The following inclusion and exclusion criteria were adopted.

Inclusion criteria

- Titles and abstracts available in the English language.
- PEth used for detecting alcohol consumption during pregnancy.
- PEth quantified in liquid human blood or in dried blood spots through liquid chromatography coupled to mass spectrometry.
- Full text in the English language.

Exclusion criteria

E. Opinion papers, editorials, and narrative reviews without novel data.

Papers not fulfilling at least one of the A–D criteria and fulfilling the exclusion criterium E were excluded. In case of doubtful classification based on title and abstract, the full text was retrieved. Any discrepancy in paper selection process was settled by consensus discussion performed by four authors (LF, AC, MP and GV).

Data extraction was conducted independently by three authors (LF, CG and GC) and the data extracted from the studies were collected in a table by two authors (AC and MP), while another author (GV) verified the accuracy of the data extraction process, in order to minimize subjective judgment. The following items were collected from each study: authors, journal and year, features of the study (type of study, subjects involved, main aims, clinical setting, and inclusion and exclusion criteria), characteristics of the investigated population (numbers of subjects and race/ethnicity), methods for estimating alcohol use, analytical method used for PEth analysis, type of measured PEth and concentration, type of collected sample, timing of collection, and main results obtained (sensitivity, specificity, positive predictive value, and negative predictive value). Any discrepancies in the data extraction process were settled by consensus discussion performed by four authors (LF, AC, MP and GV).

## 3. Results and Discussion

**Figure 1:**
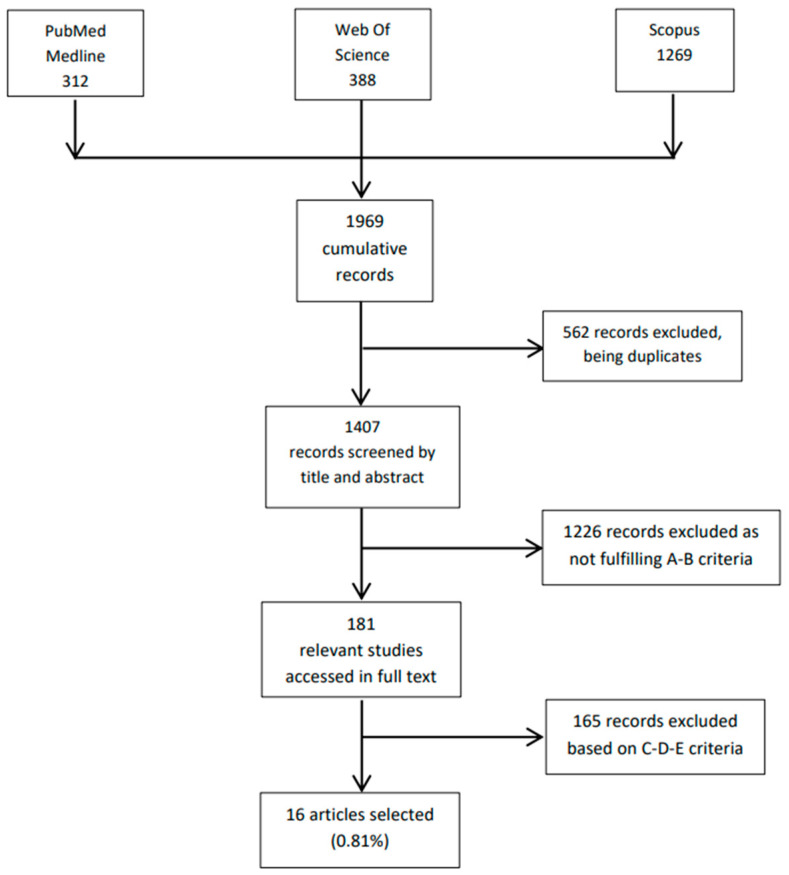
PRISMA flow-chart.

As reported in the PRISMA flow-chart (Figure 1), the combined search on the databases PubMed, Web of Science, and Scopus retrieved 1969 records, 562 of which were eliminated being duplicates. Of the 1407 records screened by title and abstract, 1226 were excluded based on criteria A and B. The remaining 181 papers were analyzed in full text with 165 records excluded based on criteria C, D, and E. Sixteen (16) papers (0.81% of the total records) were included in the present review.

The data extracted from the sixteen (16) included papers are presented in detail in Table 1.

All the included papers were original articles, of which there were seven prospective cohort/longitudinal studies [1,7,13,15,16,18,20], six cross-sectional studies [17,21,22,23,24,25], one retrospective study [26], and two observational-descriptive studies [27,28] (Table 1).

Although the search was extended to the last 20 years, only papers published in the last decade fulfilled all the inclusion criteria and were therefore analyzed in this review; in particular, it was noticed that half of the included articles (8 out of 16) were published in the last 4 years [15,17,18,22,23,24,26,27]. This result reflects the growing interest of the scientific community in PEth as a potential marker of alcohol use during pregnancy.

**Table 1:** Data extracted from the sixteen (16) included papers.

Authors	Journal and Year	Type of Study	Subjects of the Study	Aim of the Study	Clinical Setting	Inclusion/Exclusion Criteria, Subject Stratification, Types of Cases and Controls	Number of Subjects, Mean Age	Race/Ethnicity	Methods for Estimating Alcohol Use. Reported Alcohol Use	Type of Samples and Timing of Collection	Measured PEth and Mean Concentration	Analytical Method, LOQ or Cut-Off	Main Results and Conclusions
Bakhireva et al. [1]	Alcoholism: Clinical and Experimental Research 2014	Prospective cohort/longitudinal study	Mothers and newborns	To examinate validity of maternal PEth and neonatal PEth-DBS for the identification of PAE To assess the sensitivity and specificity of PEth for the detection of PAE	Pregnant women recruited at the University of New Mexico Clinic and followed to early postpartum period	Inclusion criteria: - consent in English or Spanish - singleton pregnancy - gestational age < 32 weeks Case group (PAE group): - ≥3 drinks/weeks at enrollment - at least 1 episode of binge drinking at enrollment Control group: - ≤2 drinks/weeks in periconceptional period - no binge drinking episodes in the periconceptional period - zero ounces of absolute alcohol per day and per drinking day at enrollment	PAE group: 28 newborns and 28 women 27 years ± 5.9 Control group: 32 newborns and 32 women 26.3 years ± 4.5	PAE group: - 78.6% White - 7.1% American Indian Control group: - 90.6% White - 6.3% American Indian	TFLB AUDIT	Mothers: Whole blood collected at baseline visit (mean gestational age: PAE group = 20.5 weeks ± 6.3; controls = 20.5 weeks ± 8.1) and at follow-up visit (at delivery) Newborns: Blood DBS collected at birth	16:0/18:0	LC-MS/MS LOD = 2 ng/mL LOQ = 8 ng/mL	- Maternal PEth: sensitivity 22.2%, specificity 100% - Newborn PEth DBS: sensitivity 32.1%, specificity 100% - Assessment of maternal direct ethanol metabolites (UEtG, UEtS, and PEth) combined with newborn PEth-DBS increases sensitivity to 50% without a substantial drop in specificity (93.8%)
Bakhireva et al. [25]	Alcoholism: Clinical and Experimental Research 2017	Cross-sectional study	Newborns	To estimate the prevalence of PAE in Texas by measuring PEth in infant residual DBS	Neonatal residual DBS stored in the Texas Newborn Screening Repository	Inclusion criteria: - DBS collected for newborn screening - DBS stored for ≤2 months at room temperature - DBS from diverse racial/ethnic groups and both sexes - DBS from each public health region, proportional to the birth rate	1000 residual DBS cards	- 47.8% non-Hispanic White - 40.8% Hispanic - 6.6% African American - 4.8% Asian	-	DBS cards collected within 48 h of delivery	16:0/18:1 10.2 ± 37.1 ng/mL	LC-MS/MS LOD = 2.0 ng/mL LOQ = 8.0 ng/mL PAE cut-off = 20 ng/mL	- 44.5% and 24.7% of the samples had PEth values above the LOD and LOQ, respectively - PAE prevalence: 8.4% (Cut-off = 20 ng/mL); 6.3% (Cut-off = 28 ng/mL); 1.7% (Cut-off = 100 ng/mL) - Estimated prevalence rates should be interpreted as indicative of “any” alcohol exposure approximately a month within delivery, rather than a specific level of PAE
Baldwin et al. [22]	Alcoholism: Clinical and Experimental Research 2020	Cross-sectional study	Mothers and newborns	To compare PEth levels in postpartum women and their newborn infants in Montevideo, Uruguay, and Sao Paulo, Brazil.	Pregnant women admitted to the maternity hospitals in Montevideo, Uruguay, and in Sao Paulo, Brazil	Inclusion criteria: - mother age > 18 years Exclusion criteria: - infants born with serious life-threatening birth defects	- 611 pregnant - women and 611 newborns (Uruguay) - 524 pregnant - women and 524 newborns (Brazil) 27.56 years	- 48.6% white - 35.2% mixed race - 4.5% indigenous racial identity	Thirty-minutes interview (No data relative to quantity and frequency of alcohol consumption available) Prevalence of alcohol use: - 48% (before pregnancy) - 32% (first trimester) - 12.3% (second trimester) - 13.2% (third trimester)	DBS from whole blood (mothers) collected during pregnancy DBS from heel stick (newborns), both collected within 48 h of delivery	Palmitoyl/oleoyl (16:0/18:0) Uruguay: - mothers: 43.64 ng/mL - newborns: 77.3 ng/mL Brasil: - mothers: 31.04 ng/mL - newborns: 62.2 ng/mL	LC-MS/MS LOD = 2 ng/mL LOQ (cut-off) = 8 ng/ml	- Maternal PEth above LOQ: 45.9% Uruguay, 33.3% Brasil - Neonatal PEth positive samples: 86,7% Uruguay, 76.9% Brazil - Mean PEth concentrations in newborns were significantly higher than the maternal samples - Natal sex, APGAR scores, birthweight, birth length, and head circumference were not significantly different between infants with negative or positive PEth values
Baldwin et al. [21]	International Journal of Alcohol and Drug Research 2015	Cross-sectional study	Newborns	To analyze the efficacy of screening banked newborn DBS for detection of PEth performing a retrospective assessment of statewide prevalence rates of alcohol consumption in late pregnancy that results in risky prenatal alcohol exposure To investigate the stability of PEth in stored DBS Cards	Stored residual DBS specimens collected for routine metabolic screening from the general population	Inclusion criteria: - DBS samples stored at room temperature Case-controls: - PEth were determined from at least four samples at each time point	250 deidentified DBS cards	-	US surveys relying on maternal self-report (BRFSS, NSDUH, PRAMS)	DBS cards collected at birth	16:0/18:0	LC-MS/MS LOD = 2 ng/mL LOQ = 8 ng/ml	- Storage of DBS cards at room temperature is a suitable environment for maintaining relative PEth stability for storage periods of up to six months - Stability of PEth decreases after six months at room temperature (loss of 34% of initially measured concentration) - Storage of DBS cards at −20 °C improves PEth stability and the PEth detection in stored samples versus storage at room temperature for 30 days - DBS positive specimens: 4% - In other 23 DBS specimens, PEth concentrations were above LOD
Bracero et al. [16]	Reproductive Toxicology 2017	Prospective cohort/longitudinal study	Mothers	To compare rates of alcohol use between urine ethanol testing and self-reporting (Method 1) and Phosphatidylethanol (PEth) dried blood spot testing and self-reporting (method 2)	Pregnant women attending the prenatal care medical center between July 2013 and March 2014	Inclusion criteria: - availability of a complete medical history - availability of alcohol laboratory screen testing - continued attendance to the prenatal clinic	314 pregnant women. 24.9 years ± 5.8	- White (82.2%) - Black/African American (16.2%) - Asian (0.6%) - Native American (0.6%) - Bi-racial (0.6%)	ACOG prenatal record questionnaire	DBS from blood specimens, collected during first trimester (mean gestational age: 11.3 ± 7.3 weeks)	Palmitoyl/oleoyl (16:0/18:0)	LC-MS/MS LOD = 2 ng/mL LOQ = 8 ng/ml	- Method 1 identified 11 patients with alcohol use (5 urine and 6 self-reported), while method 2 - identified 28 (22 PEth and 6 self-reported). One patient had both a positive urine and PEth - In 32 patients, alcohol use was detected using all methods - Self-reporting and PEth testing had an absolute increase rate of 5.4% in identifying women who used alcohol during pregnancy - PEth was significantly better at detecting alcohol use than urine ethanol - PEth appears to capture alcohol drinkers that are not being identified by the ethanol urine screen test
Breunis et al. [18]	BMC Pregnancy and Childbirth 2021	Prospective cohort/longitudinal study, cross-sectional, single center study	Mothers	To evaluate biochemically assessed prevalence of alcohol consumption during early pregnancy using PEth levels.	Pregnant women who were under the care of the department of the Erasmus MC between September 2016 and October 2017	Inclusion criteria: - gestational week < 15 weeks Exclusion criteria: - gestational week > 15 weeks - unknown gestational age - no routine laboratory analysis - age < 18 years	684 pregnant women. 31.7 years (SD 4.9)	-	Self-reported consumption	Whole blood collected at gestational week < 15	16:0/18:1 (POPEth) 16.0/18.2 (PLPEth) 18.1/18.1 (DOPEth)	LC-MS/MS LOD = 2.0 µg/L (16:0/18:1) 2.0 µg/L (16:0/18:2) 2.0 µg/L (18:1/18:1) LOQ = 6.0 µg/L (16:0/18:1) 6.0 µg/L (16:0/18:2) 3.0 µg/L (18:1/18:1)	- 5.3% pregnant women had blood PEth above LOQ - The mean week of gestation of women with a positive PEth test was 9.6 weeks (SD 1.9). Of these women, 11% reported alcohol consumption to their obstetric care provider - 44.4% of positive PEth tests had at least one value below the LOQ but above the LOD - 0.3% of women reported alcohol consumption despite a negative PEth test - Age, week of gestation, gravida, parity, smoking, and country of birth were not significantly associated with a positive PEth test
Comasco et al. [7]	Alcoholism: Clinical and Experimental Research 2012	Prospective cohort/longitudinal study	Mothers	To evaluate methods to assess maternal drinking during pregnancy—To investigate possible influences of PAE	Women attending the maternity clinic at Uppsala University Hospital between October 2007 and May 2009	Inclusion criteria: - volunteer pregnant women. Subject stratification: - 16 samples from women with AUDIT score ≥ 9 before pregnancy - 42 samples from women with AUDIT-C score ≥ 1 at 32 weeks - 19 samples from women with AUDIT-C - score = 0 at week 32	77 random blood samples from 2264 pregnant women30.4 years (17–49)	-	AUDIT (16–18 weeks of gestation) for alcohol use before pregnancy AUDIT-C (32 weeks of gestation) for alcohol use during pregnancy	Whole blood collected at 16–18 weeks of gestation	-	LC-MS/MS Cut-off (reporting limit) = 0.1 µmol/L	- All PETH values were below reporting limit, while AUDIT suggested that a significant number of women continued to consume alcohol during pregnancy - The birthweight of female newborns was related to PAE (p = 0.019)
Di Battista et al. [26]	Alcoholism: Clinical and Experimental Research 2022	Retrospective Study	Newborns	To estimate rates of prenatal alcohol exposure (PAE)	Random selection of 2011 residual DBS collected over a 1-week time period.	Inclusion criteria: - C ontain enough blood to take three punches - Do not have received a blood transfusion prior to sampling	2006 residual DBS	-	-	-	16:0/18:1 16:0/18:2 16:0/16:0 18:0/18:2 18:1/18:1 18:0/18:1 16:0/20:4 311 samples tested positive (16:0/18:1 > 20 ng/mL). 24 samples had a value > 284 nM. Main value in Peth—Positive samples 16:0/18:1 = 103 ± 173 nM 16:0/18:2 = 73.5 ± 130 nM 16:0/16:0 = 17.9 ± 18.5 nM 18:0/18:2 = 10.6 ± 19.7 nM 18:1/18:1 = 9.93 ± 17.0 nM 18:0/18:1 = 16.6 ± 24.0 nM 16:0/20:4 = 86.1 ± 120 nM Total Peth= 318 ± 478 nM	LC-MS/MS LOD = 2 nM for all Peths, with the exception of 16:0/18:2 and 16:0/20:4 which had 4 nM. LOQ = 4 nM (16:0/16:0, 18:0/18:2, 16:0/18:1, 18:0/18:1) 8 nM (16:0/18:2, 18:1/18:1, 16:0/20:4)	- Rate of late-pregnancy PAE in Ontario is 15.5%, with 1% showing levels coherent with heavy consumption. - Of the six Peth homologues quantified in addition to 16:0/18:1, 16:0/20:4, and 16:0/18:2 were the most abundant (80% of the total PEth). - There is considerable individual variation in each PEth with CV ranging from 25.6% (16:0/18:1) to 94.3% (16:0/16:0) - Correlation between PEth homologues and total PEth were generally moderate to strong, with the exception of 16:0/16:0. - Sex showed no association with PAE status. LBW and PT were associated with increased odds of a positive PAE test result. SGAs had a lower risk of PAE.
Finanger et al. [27]	Alcoholism: Clinical and Experimental Research 2021	Observational descriptive study	Mothers	Investigate the prevalence of positive PEth values as an indicator of early prenatal alcohol exposure in pregnant women	Rhesus type and antibody screening in pregnant women attending the Clinic between September 2017 and October 2018	Inclusion criteria: - all blood samples collected for Rh screening Exclusion criteria: - women in the reservation register - age < 18 years or > 50 years - samples with insufficient amount of blood or technical factors Subject stratification: - 3.451 (76.1%) samples from first trimester - 830 (18.3%) samples from second trimester - 252 (5.6%) samples of unknown timing	4.533 whole blood samples from 4.067 pregnant women Women with PEth positive sample: 30.3 years ± 5.5 Women with PEth negative sample: 30.2 years ± 4.7	-	-	Whole blood collected at gestational week 12 and 24	16:0/18:1 0.026 µM (0.003-0.287)	UPLC-MSMS LOQ = 0.003 µM	Fifty-eight women had a positive PEth sample collected during pregnancy: first trimester 50; second trimester 3; 5 unknown timing.
Kwak et al. [13]	Clinical Toxicology 2012	Prospective cohort/longitudinal study	Mothers	To evaluate PEth concentrations in pregnant women with positive history of low-to-moderate alcohol ingestion	Pregnant women referred for teratogen-risk counseling because of recent history of alcohol ingestion	Inclusion criteria: - volunteer pregnant women Control group: - 26 first-trimester pregnant women reporting no alcohol consumption for at least 6 months before conception	13 first-trimester pregnant women Case group: 32.3 years ± 5Control group: 32.4 years ± 4	-	Self-reported consumption 7.5 (2.5–20) drinks/week	Whole blood collected during firsttrimester of gestation	16:0/16:0 16:0/18:1 18:1/18:1 PETh 16:0/16:0 = 10.6 nmol/L (1.2–25.3) PETh 16:0/18:1 = 47.8 nmol/L (3.5–177.0) PEth 18:1/18:1 = 3.2 nmol/L (0.2–10.2)	LC-MS/MS LOD = 0.4 nmol/L (16:0/16:0) 0.9 nmol/L (16:0/18:1) 0.4 nmol/L (18:1/18:1) LOQ = 1.5 nmol/L (16:0/16:0) 3.1 nmol/L (16:0/18:1) 1.2 nmol/L (18:1/18:1)	In all cases, PEth 16:0/18:1 levels were above LOQ, whilst PEth 16:0/16:0 and 18:1/18:1 were below LOQ in two and six subjects, respectively.
Kwak et al. [20]	Clinical Toxicology 2014	Prospective cohort/longitudinal study	Mothers	To characterize PEth blood concentrations to differentiate different levels of alcohol exposure in pregnant women	Pregnant women referred to the Clinic for antenatal care	Inclusion criteria: - written informed consent Exclusion criteria: - psychiatric problems or cognitive impairment Subject stratification: - abstainers 0 drinks/week - light drinkers ≤ 3 drinks/week - moderate drinkers 3–7 drinks/week - heavy drinkers ≥7 drinks/week	305 first-trimester pregnant women Abstainers 32.7 years ± 3.8 Light drinkers 32.3 years ± 4.0 Moderate drinkers 33.2 years ± 4.4 Heavy drinkers31.9 years ±4.9	-	Self-reported consumption	Whole blood collected within 3–4 weeks after recruitment (mean gestational age: 6.9–7.8 weeks)	16:0/16:0 16:0/18:1 18:1/18:1	LC-MS/MS LOD = 0.4 nmol/L (16:0/16:0) 0.9 nmol/L (16:0/18:1) 0.4 nmol/L (18:1/18:1) LOQ = 1.5 nmol/L (16:0/16:0) 3.1 nmol/L (16:0/18:1) 1.2 nmol/L (18:1/18:1)	- PEth resulted above LOQ in 4.8% of abstainers, in 26.4% of light drinkers, in 54.5% of moderate drinkers, and in 100% of heavy drinkers - PETh concentrations were significantly correlated to drinks per occasion and days drinking per week - PEth concentration increased by 9.5 nmol/L per drink ingested on each occasion, and by 5.8 nmol/L per drinking/day/week
Maxwell et al. [23]	Reproductive Toxicology 2019	Cross-sectional study	Newborns	To detect PAE assessing biomarkers	DBS collected between January 2013 and February 2015	Exclusion criteria: - improper collection of the sample	162 newborns	Positive PETh testing: - White 36 (83.7%) - Black 7 (16.3%) Negative PETh testing: - White 94 (79.0%) - Black 23 (19.3%) - Other 2 (1.7%)	-	DBS from umbilical cord collected immediately after birth	Palmitoyl/oleoyl (16:0/18:0)	LC-MS/MS LOQ = 8 ng/mL	- PEth tested above LOQ in 26.5% samples - PAE was non associated with neonatal dysmorphic features or short-term adverse outcomes
Raggio et al. [15]	Journal of Acquired Immune Deficiency Syndromes 2019	Prospective cohort/longitudinal study	Mothers	To investigate alcohol use and under-reporting of alcohol use in pregnant women HIV-positive in South Africa and Uganda	Women attending the outpatient clinics offering HIV care in Uganda and South Africa	Inclusion criteria: - HIV-positive women - ART naive or initiated within one month - age ≥ 18 years - living within 60 km - intention to stay in the area for the next year - if pregnant, no more than 34 weeks - English or local speakers - informed consent Exclusion criteria: - CD4 at enrollment = 200–349 - CD4 at enrollment < 200 (pregnant women only) Case group: - pregnant women - Control group: - non-pregnant women	163 pregnant women 255 non-pregnant women 29 years (24–35)	-	AUDIT-C	DBS from venous blood draws	-	LC-MS/MS LOQ = 8 ng/mL	- 36.2% pregnant and 38.8% non-pregnant women had PETH above LOQ - 39.9% pregnant and 44.3% non-pregnant women had PEth above LOQ and/or AUDIT-C > 0. - 16.0% pregnant and 12.9% non-pregnant women had PETH ≥ 8 ng/mL and AUDIT-C = 0 [underreport of any alcohol use] - 23.3% pregnant and 29.4% non-pregnant women had PETH ≥ 50 ng/mL and/or AUDIT-C - ≥3 [heavy/hazardous alcohol use] - Alcohol use was prevalent and under-reported among pregnant HIV in South Africa and Uganda, with similar rates demonstrated by pregnant and non-pregnant women

### 3.1. Main Aims of the Included Studies

All the included studies, although with slightly different secondary objectives, had a main aim of the identification of alcohol consumption during pregnancy. Eight out of the sixteen included papers assessed maternal alcohol consumption during pregnancy; Di Battista et al. [26], for example, analyzed PEth in neonatal blood without any comparison with the mother’s declaration on alcohol intake during pregnancy.

### 3.2. Reported Alcohol Intake

In three included studies, the reported daily alcohol intake of the mother was compared not only with the maternal blood PEth concentration, but also with the neonatal blood PEth concentration after birth [1,7,22].

In 5 of the 16 included records, the assessment of alcohol consumption during pregnancy was performed using a standardized questionnaire. Four different types of questionnaires were employed:

- Alcohol Use Disorders Identification Test (AUDIT) [1,7,15];
- Timeline Follow-back (TFLB) [1];
- American College of Obstetrician and Gynecologist Prenatal Record Questionnaire (ACOG questionnaires) [16];
- Tolerance, Worry about drinking, Eye-opener, Amnesia, and Cut down on drinking (TWEAK test) [17].

In three included records, only self-reporting, without any structured questionnaires, has been employed for reconstructing maternal alcohol exposure during pregnancy [13,18,20].

Although misreporting or under-reporting due to shame or other social reasons always remains an issue, employing standardized questionnaires should help in minimizing these problems, and could increase the comparability of the collected data. The high heterogeneity encountered in the included records suggests an urgent need for an international effort of standardization, with the hopeful creation of a dedicated structured questionnaire for collecting alcohol use/abuse data during pregnancy.

### 3.3. Isoforms of PEth

As well-known, numerous molecular species of PEth can be detected and quantified in blood by liquid chromatography coupled to mass spectrometry with a lack of consensus in the scientific community on their diagnostic efficiency in different clinical and forensic settings. In recent years, PEth 16:0/18:1 and PEth 16:0/18:0 have been increasingly used for the identification of chronic alcohol abuse or dependence and for monitoring abstinence.

**Figure 2:**
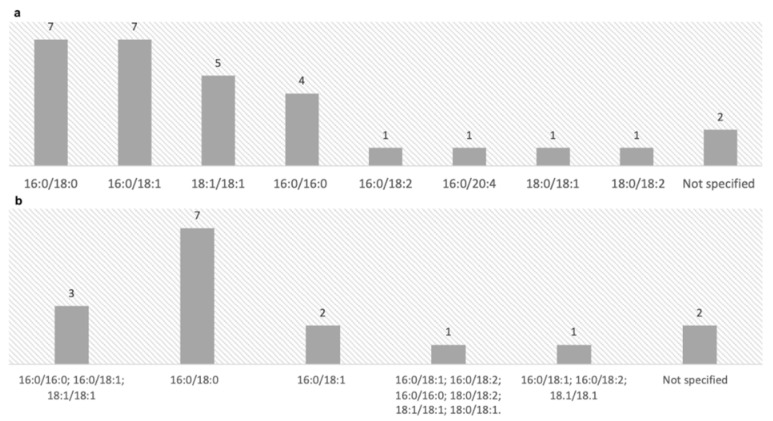
(**a**) Frequency of PEth isoforms quantified. (**b**) Combinations of PEth isoforms quantified in the 16 included studies.

In the included records (Figure 2), the following major isoforms have been quantified:

- PEth 16:0/18:0 [1,16,17,21,22,23,24];
- PEth 16:0/18:1 [13,18,20,25,26,27,28];
- PEth 16:0/16:0 and 18:1/18:1 [13,20,26,28].

### 3.4. Units of Measurement

Regarding the different units of measurement of PEth molecular species, the 1990–2010 literature generally used moles, whereas more recent studies (2010–2022) favored the use of fractions of weight over volume (e.g., ng/mL or µg/L).

In the present review, it was noticed that 10 included studies expressed PEth concentrations in weight over volume fractions [1,15,16,17,18,21,22,23,24,25], whereas 6 papers used moles [7,13,20,26,27,28]. The latter unit of measurement requires the knowledge of the molecular weight of each quantified PEth isoform, making it difficult to compare studies that utilize different molecular species and impossible to convert total PEth concentration without detailed information on the concentration of each isoform.

### 3.5. Interpretative Cut-Offs and Sensitivity/Specificity of PEth

Another issue regards the identification of an interpretative cut-off or threshold capable of discriminating with high selectivity an active alcohol intake during pregnancy (i.e., ingestion of alcoholic beverages) from an unintentional exposure to minute amounts of ethanol contained, for example, in food (e.g., cakes with liquor and fermentation of fruit) [29,30,31].

In the included studies, most authors interpreted total PEth concentration basing on the analytical threshold (i.e., lower limit of quantitation), generally set at 8 ng/mL [1,15,16,17,21,22,23,24,25]. Only one record [25] proposed an interpretative threshold (total PEth > 20 ng/mL) for identifying alcohol exposure during pregnancy, but no detailed data on sensitivity and specificity were presented.

Therefore, in contrast to the general population where several authors proposed different PEth thresholds to differentiate teetotalers from social or heavy drinkers, in this specific field of research there is still a lack of studies examining interpretative PEth cut-offs to discriminate if and how much a woman drinks ethanol during pregnancy.

Consequently, the diagnostic efficiency in terms of sensitivity and specificity for detecting and quantifying alcohol use during pregnancy has still to be systematically assessed. In the included studies, only Bakhireva et al. [1] reported that PEth exhibited low sensitivity (22.2%) and high specificity (100%) even for low alcohol exposures during pregnancy.

### 3.6. Biological Matrices Involved and Categorization of the Studies

All the included studies assayed PEth in at least one biological matrix; seven (7) studies [7,13,15,16,18,20,27] quantified PEth in maternal blood, seven studies [17,21,23,24,25,26,28] in newborn blood, and only two studies [1,22] in both maternal and neonatal blood.

Hence, to better analyze the extracted data, the included records were divided into three categories:

- PEth in maternal blood [7,13,15,16,18,20,27];
- PEth in neonatal blood [17,21,23,24,25,26,28];
- PEth in both maternal and neonatal blood [1,22].

#### 3.6.1. PEth in Maternal Blood

In the 7 (out of 16) papers included in this section [7,13,15,16,18,20,27], a total of 5882 pregnant women underwent PEth analysis in blood during pregnancy.

In six [7,13,16,18,20,27] out of seven papers, PEth quantification was performed in maternal blood collected from pregnant women attending to an antenatal care center; differently, Raggio et al. [15] examined pregnant and unpregnant women referring for HIV therapy.

Blood samples were not collected at the same time of pregnancy in all the included records:

- in three records sample collection was performed during the first trimester [13,16,20];
- in one record before the 15th week of gestational age [18];
- in one record between the 16th–18th week of gestational age [7];
- in one record two blood withdrawals were performed [27]; the first at the 12th week of gestational age and the second one at the 24th week of gestational age;
- in one record before the 34th week of gestational age [15].

In five records, PEth was quantified in liquid venous blood [7,13,18,20,27], whereas in two records, it was performed in DBSs [15,16].

Only in two out of the seven records analyzed here, populations were stratified into subgroups on the basis of the women’s self-reported alcohol consumption before and during pregnancy [7,20]. In detail, Comasco et al. [7] administered the same questionnaire (AUDIT) in two different time of pregnancy: the first regarded pre-pregnancy alcohol consumption, administered between the 16th and the 18th gestational week, and the second one regarded alcohol consumption during pregnancy, being administered at the 32nd gestational week. In all cases, PEth was under the cut-off of 0.1 μmol/L, while the AUDIT score suggested that a significant number of women continued consuming alcohol during pregnancy. Another type of stratification, based on weekly alcohol self-report (A.U./week), was performed by Kwak et al. [20], where the population involved was divided into teetotalers (0 A.U./week), light drinkers (≤3 A.U./week), moderate drinkers (3–7 A.U./week), and heavy drinkers (≥7 A.U./week). The authors concluded that all women (100%) who declared a heavy alcohol consumption (≥7 A.U./week) had PEth concentrations above the LOQ (limit of quantification); PEth sensitivity proportionally decreased with the reduction of the declared alcohol intake.

Breunis et al. [18] found that 44.4% of the cases with a positive blood PEth result exhibited a PEth concentration above the lower limit of detection (LOD) but below the lower limit of quantitation (LOQ). These results may suggest that a further improvement of the analytical performance of the methods used for blood PEth quantitation could favor an improvement of the diagnostic sensitivity of PEth in maternal blood for detecting an active alcohol exposure during pregnancy.

Nowadays, despite that the employment of PEth for monitoring alcohol consumption in pregnancy still has numerous limitations and the data obtained until now are incomplete, some authors have already begun to use PEth as a screening tool in the clinical practice; in detail, a study performed in Norway [27] analyzed blood PEth concentration in the same samples collected for Rhesus typing, which is routinely performed in all pregnancies around the 12th gestational week. In the above study, the authors found out that 1.4% of the included cases had a positive PEth sample around the 12th gestational week, whereas only 0.4% of the included cases had a positive sample around the 24th gestational week.

#### 3.6.2. PEth in Neonatal Blood

In the United States, 2–5% of school-aged children are estimated to be affected by FASD, but the prevalence of PAE might be substantially underreported [32]; therefore, an accurate detection of PAE in newborns might offer the opportunity for an early identification of children at risk for future neurocognitive disorders, allowing for an early intervention to prevent or reduce long-term consequences.

Besides other direct ethanol metabolites measured in the meconium (e.g., FAEEs, EtG and EtS [33,34]), in recent years, PEth has been proposed as a very promising biomarker of PAE, being measurable both in whole venous blood and DBS.

All the included seven papers regarding PEth in neonatal blood have used DBS for blood collection. DBS can be obtained from venous or capillary blood, being minimally invasive, easy, and cheap to collect, storage, and transport, giving the opportunity of a potential integration with routine newborn screening for metabolic diseases [1,22].

In one of the included papers [23], PEth was quantified in blood collected from the umbilical cord immediately after birth, whereas the other six papers used DBS from heel capillary blood [17,21,24,25,26,28].

Unfortunately, only two out of the seven papers belonging to this section provided data on maternal alcohol exposure; Baldwin et al. [21] did not correlate neonatal blood PEth concentration to the maternal alcohol intake during pregnancy, whereas Stevens et al. [17] highlighted that about 60% of the included neonates, which were born from women exposed to ethanol during pregnancy, had a capillary blood PEth concentration above the analytical threshold (8 ng/mL). Therefore, none of the included papers tested the diagnostic efficiency of PEth in neonatal blood to reconstruct the alcohol consumption of the mother during pregnancy.

Umer et al. [24] and Yang et al. [28] reported a potential correlation between high neonatal blood PEth levels and low birth weight, preterm birth, and increased risk of miscarriage, although these observations did not reach any statistical significance.

Stevens et al. [17] reported that, if the pregnancy is unplanned, there is an increased risk of moderate to heavy alcohol exposure in the early stages of pregnancy, as women became aware of their pregnancy later.

#### 3.6.3. PEth in Both Maternal and Neonatal Blood

Two (2) out of the sixteen included papers quantified PEth in both maternal and neonatal blood.

Bakhireva et al. [1] divided the included pregnant women into two groups (i.e., moderate alcohol consumption versus low/absent alcohol intake) based on their AUDIT scores and on the in-depth TFLB calendar assessments and compared total PEth concentration in maternal venous blood to total PEth concentration in neonatal DBS. Neonatal PEth demonstrated a higher sensitivity (32.1%) than maternal PEth (22.2%) for detecting moderate alcohol consumption.

Baldwin et al. [22] included 611 pregnant women from Uruguay and 524 from Brazil and compared maternal to neonatal blood PEth concentrations.

The authors found out that the infants had significantly higher blood PEth concentrations than their mothers. This phenomenon has not yet been explained and more studies are needed to understand its molecular origin. A possible explanation could be that the ethanol ingested by the mother, crossing the placenta and reaching blood concentrations similar to the maternal ones, accumulates in the amniotic fluid (due to its slow turnover) with a consequent longer exposure of fetal red blood cells to alcohol and a higher production of PEth [4].

Only little evidence has been published on neonatal blood PEth concentrations and, at the present time, it is unclear if dosing PEth both in maternal and neonatal blood could enhance the diagnostic efficiency of the marker for detecting an alcohol exposure during pregnancy.

## 4. Conclusions

Alcohol consumption during pregnancy, even at low doses, may damage the fetus. Pregnant women tend to underreport their alcohol consumption out of shame or social stigma generating the need for sensitive and specific biomarkers capable of identifying any alcohol use during pregnancy in order to promote educational programs and counselling interventions.

In the recent literature, among the proposed biomarkers, PEth has emerged due to its high specificity and possibility to be quantified in both maternal and neonatal blood, also using dried blood spots (DBS) collected for routine screenings.

As reported in the present review, only few studies (16), where ethanol exposure during pregnancy was not always collected through structured questionnaires, and with a non-homogeneous stratification of the included populations, have been published on this topic until now.

In several included papers, PEth has proven more sensitive than self-reports for identifying pregnant women with an active alcohol intake with the diagnostic efficiency of the marker improving with the increase of the maternal alcohol intake. Probably, a further improvement of PEth diagnostic efficiency might be reached by improving the analytical performance of the methods used for its quantitation in blood (i.e., liquid chromatography coupled to mass spectrometry). Moreover, in order to implement PEth analysis in the assessment of PAE, more data about a worldwide established interpretative cut-off, its certain sensitivity and specificity, and the diagnostic efficiency of the different isoforms are still needed.

Further studies, performed on wider and well-stratified populations, are still needed to verify if PEth concentration in maternal and/or neonatal blood could be capable of identifying even minimal ethanol intakes during the first trimester.
